# The impact of psychological factors on recovery from injury: a multicentre cohort study

**DOI:** 10.1007/s00127-016-1299-z

**Published:** 2016-11-01

**Authors:** Blerina Kellezi, C. Coupland, R. Morriss, K. Beckett, S. Joseph, J. Barnes, N. Christie, J. Sleney, D. Kendrick

**Affiliations:** 1 Division of Psychology, School of Social Sciences, Nottingham Trent University, Burton Street, Nottingham, NG1 4BU UK; 2 Division of Primary Care, University of Nottingham, Floor 13, Tower Building, University Park, Nottingham, NG7 2RD UK; 3 Division of Psychiatry and Applied Psychology, Institute of Mental Health and CLAHRC East Midlands, University of Nottingham, Nottingham, UK; 4 Education Centre, University of the West of England, Research and Innovation, University Hospitals Bristol NHS Foundation Trust, Upper Maudlin Street, Bristol, BS2 8AE UK; 5 School of Education, University of Nottingham, Nottingham, NG8 1BB UK; 6 Design School, Loughborough University, Ashby Road, Loughborough, LE11 3TU UK; 7 Department of Sociology, University of Surrey, Guildford, Surrey GU2 7XH UK; 8 Centre for Transport Studies, UCL, Gower Street, London, WC1E 6BT UK

**Keywords:** Unintentional injury, Recovery, Depression, Psychological, Longitudinal

## Abstract

**Purpose:**

Unintentional injuries have a significant long-term health impact in working age adults. Depression, anxiety and post-traumatic stress disorder are common post-injury, but their impact on self-reported recovery has not been investigated in general injury populations. This study investigated the role of psychological predictors 1 month post-injury in subsequent self-reported recovery from injury in working-aged adults.

**Methods:**

A multicentre cohort study was conducted of 668 unintentionally injured adults admitted to five UK hospitals followed up at 1, 2, 4 and 12 months post-injury. Logistic regression explored relationships between psychological morbidity 1 month post-injury and self-reported recovery 12 months post-injury, adjusting for health, demographic, injury and socio-legal factors. Multiple imputations were used to impute missing values.

**Results:**

A total of 668 adults participated at baseline, 77% followed up at 1 month and 63% at 12 months, of whom 383 (57%) were included in the main analysis. Multiple imputation analysis included all 668 participants. Increasing levels of depression scores and increasing levels of pain at 1 month and an increasing number of nights in hospital were associated with significantly reduced odds of recovery at 12 months, adjusting for age, sex, centre, employment and deprivation. The findings were similar in the multiple imputation analysis, except that pain had borderline statistical significance.

**Conclusions:**

Depression 1 month post-injury is an important predictor of recovery, but other factors, especially pain and nights spent in hospital, also predict recovery. Identifying and managing depression and providing adequate pain control are essential in clinical care post-injury.

**Electronic supplementary material:**

The online version of this article (doi:10.1007/s00127-016-1299-z) contains supplementary material, which is available to authorized users.

## Introduction

Unintentional injuries can have a significant impact on health services and individuals’ physical and psychological health. They are estimated to account for 9% of disability-adjusted life years globally [1] and nearly 700,000 hospital admissions in England yearly [2]. A significant proportion of people do not fully recover 12 months after injury [3] including those with less severe injuries [4]. Many factors have been associated with poorer recovery, including pre-injury health status, age, gender, admission status, injury severity, body region, place of injury, pain, psychological morbidity, working status post-injury and insurance status [3, 5–9]. The individual variation in the aftermath of unintentional injuries is poorly understood partly because of the diversity of the influencing factors and the lack of an overarching model that brings these variables together.

Health models like the stress and coping model of Lazarus and Folkman [10] argue that the variety of responses to stressors depends on the appraisal of the stressor, i.e. the unintentional injury. According to this model, individuals actively try to appraise the potential threat of the injury to health and well-being, as well as the resources available to deal with the stressor. Where there are resources available to support the individual, then the injury would be perceived as less threatening over time. The contrary could also be true; psychological, work or financial problems, or lack of support post-injury could prolong the threat of the injury and the individual’s appraisal of its severity. This continuous reappraisal of the threat could account for variability in outcomes post-unintentional injury, including poor outcomes in those with relatively minor injuries.

Whilst injury and demographic and pre-injury health status are not modifiable, there are effective interventions for psychological factors [11]. This is particularly important given how common psychological morbidities [especially depression, anxiety and post-traumatic stress disorder (PTSD)] follow unintentional injuries. However, like other outcomes [3, 5–9], the prevalence of psychiatric morbidity following unintentional injury varies considerably between studies. A review of psychiatric morbidity following motor vehicle injury found that the rates of depression across studies ranged between 21 and 67%, anxiety 4–87% and PTSD 0–100% [12]. Another review with traumatic injury survivors found that the prevalence of depression ranged between 6 and 42%, anxiety between 4 and 24% and PTSD in most studies between 10 and 30%, [13], and a final review of general and specific injury populations found that the prevalence of PTSD ranged between 2 and 38% at 12 months [14].

Research shows that psychological morbidity predicts injury outcomes such as return to work, physical function and pain [3, 15]. For example, depression and PTSD (intrusion symptoms) shortly post-injury and at 6 months predicted poorer quality of well-being (mobility/physical activity/social activity as measured by the Quality of Well-being Scale) at 12 and 18 months post-injury [16]. Post-injury PTSD and emotional distress predicted higher pain and disability (measured by the Neck Disability Index score) 6 months post-injury among those experiencing whiplash injuries [17]. Post-injury depression predicted poorer functional outcome (limitations to work/housework/social life) at 12 months post-moderate injury [8].

Functional outcomes do not fully capture the process of recovery. There is no widely accepted definition of recovery from injury, but the following definition of recovery from mental illness could apply equally well to injuries: “a deeply personal, unique process of changing one’s attitudes, values, feelings, goals, skills and/or roles. It is a way of living a satisfying, hopeful, and contributing life even within the limitations caused by illness” [18]. The same author also argues that “recovery is a multidimensional concept: there is no single measure of recovery, but many different measures that estimate various aspects of it” [18]. Outcomes such as return to work, physical function, pain or activity correlate poorly with self-rated recovery because they overlook the individual’s social context, own understanding, appraisal and definition of recovery [19]. This is likely to be based on physical and emotional symptoms and adjustments or adaptations and reappraisals required to live with the consequences of the injury [20] and might partly explain prolonged recovery periods [21] and high levels of health service use [22] associated with some relatively minor injuries. As functional and health status measures may not fully capture the complex nature of recovery, additional outcome measures, such as participants’ perception of recovery are needed. To our knowledge., no published prospective studies have investigated the role of psychological factors in predicting self-reported recovery in adults experiencing a wide range of unintentional injuries.

The analyses presented in this paper address this research gap and also address some of the limitations of prospective injury outcome studies highlighted in recent systematic reviews [23–25]. These include use of specific injury populations as opposed to a wide range of injuries of varying severity [25, 26], small sample sizes, low response or follow-up rates or failure to adequately adjust for possible confounders [23]. The present study aims to investigate the impact of early psychological morbidity on self-reported recovery whilst controlling for a range of social, injury, physical and demographic factors.

## Methods

The methods of the Impact of Injuries Study have been described in detail in the published protocol [28]. The study had multi-centre approval from the Nottingham Research Ethics Committee 1 (number: 09/H0407/29).

### Study design

Prospective longitudinal study set in five NHS hospitals in Nottingham, Bristol, Leicester and Surrey, UK.

### Participants

Participants, aged 16–70 years, were recruited following hospital admission for a range of unintentional injuries between June 2010 and June 2012. Inclusion criteria included the (a) ability to give informed consent, (b) recruitment within 3 weeks of injury and (c) an address to enable follow-up. Those with significant head injury (loss of consciousness, amnesia or a Glasgow Coma Scale of <15 at presentation) were excluded due to difficulty distinguishing between sequelae of mild head injury and psychological morbidity [27]. Participants were recruited face to face, by post or phone. The study used quota sampling between June 2010 and May 2011. This was based on age (16–24, 25–59, 60–70), sex and injury type (12 categories) to ensure inclusion of a wide range of injuries and to allow comparison with other studies using general injury populations. This is described in further detail in the published protocol [28]. However, due to slow recruitment, all eligible patients could participate from June 2011. Clinical staff (e.g. research nurses) identified patients being potentially eligible and asked patients if they agreed to be approached about the study. Members of the research team then assessed eligibility of those agreeing to be approached.

### Data collection

Participants completed self-administered questionnaires at recruitment (baseline) and at 1, 2, 4 and 12 months post-injury. Baseline questionnaires measured socio-demographic details [age, marital status, ethnicity, number of cars in household, living alone, employment status, area-level deprivation (the Index of Multiple Deprivation (IMD) 2010)] [29]; pre-injury quality of life (EQ5D) [30], long-term health conditions, anxiety and depression [Hospital Anxiety and Depression Scale (HADS)] [31], alcohol problems [Alcohol Use Disorder Identification Test (AUDIT)] [32], substance use [Drug Abuse Screening Test (DAST)] [33], social functioning [Social Functioning Questionnaire (SFQ)] [34] and injury details. The Abbreviated Injury Scale (AIS) [35] was used to score injury severity using medical record data. Participants’ maximum injury severity across all injuries was grouped into three categories: minor (AIS = 1), moderate (AIS = 2) and serious to maximum (AIS = 3–6). Follow-up questionnaires also included self-reported recovery [36], the HADS, Impact of Events Scale (IES) to measure PTSD [37], stressful life events related to the injury [List of Threatening Events (LTE)] [38], time off work since injury, social support [Crisis Support Scale (CSS)] [39], positive and negative changes in outlook [Change in Outlook Questionnaire, (CiOQ)] [40] and legal proceedings or compensation claims due to injury. A researcher administered a structured clinical interview (SCID) [41] which measured psychiatric diagnosis at baseline for all participants and at follow-up for those scoring borderline or above on the HADS depression (>7), HADS anxiety (>7), IES (>18 for each subscale or >29 for combined scores), AUDIT (>7) and/or DAST scales (>2).

### Statistical analysis

Comparisons of baseline and 1 month characteristics were made between participants returning both 1 and 12 month questionnaires and those who did not using Chi-square tests for categorical variables and Mann–Whitney *U* tests for non-normally distributed continuous variables. We used self-reported recovery at 12 months as our outcome variable, as full recovery was rarely reported at earlier time points (see “Results”); we combined categories in the questionnaire into a binary variable for full recovery (yes/no). We compared health status (EQ5D utility index and the Health Utilities Index) between those who reported that they had fully recovered at 12 months and those who had not using Mann–Whitney *U* tests.

Clinical intervention for psychological morbidity within the first few weeks post-injury is not always indicated, so analyses used psychological morbidity variables (HADS depression, HADS anxiety, AUDIT, DAST and IES) reported at 1 month as predictors of recovery. The changes from baseline to 1 and 12 months in the proportions meeting the criteria for psychological morbidity caseness and SCID-DSM-IV criteria for mental disorder were compared using McNemar’s tests.

Odds ratios and 95% confidence intervals for full recovery at 12 months were estimated using logistic regression. The linearity of relationships between continuous variables and recovery was assessed by adding higher-order terms to models with categorisation (see Table 1) where necessary. Correlations between psychological predictors and other related predictors considered for model inclusion were assessed, and predictors with a correlation with a psychological variable above 0.5 were not considered for inclusion in the model. The model was built in steps, initially only including a priori defined confounders (study centre, age and sex) (model A). Psychological predictors measured at 1 month (HADS (depression and anxiety subscales), IES (avoidance and intrusion subscales), AUDIT and DAST) were added separately in order of significance in univariate analyses. Only psychological predictors with a likelihood ratio test (LRT) *p* value of <0.05 were retained in the model (model B). Potential predictors of recovery measured at baseline (number of prior psychiatric morbidities, HADS (depression and anxiety subscales), AUDIT, DAST, prior long-standing illness, work status, ethnic group, marital status, deprivation, length of hospital stay, injury severity, number of injuries, body part injury, injury mechanism and place of injury) were added in one block and removed in order of least statistical significance first based on the LRT (*p* ≥ 0.05). Those with a *p* value of ≥0.05 whose removal changed odds ratios for any of the significant 1 month psychological predictors by more than 10% were retained in the model (model C). Finally, other potential predictors measured at 1 month post-injury (pain, social support, life events, compensation and litigation) were added in one block and tested for removal as above (model D). We tested for interactions between psychological predictors and other variables included in model D by adding interaction terms (*p* < 0.01) to the model. Collinearity between variables in the final model was assessed by examining the covariance correlation matrix and estimating variance inflation factors.

**Table 1:** Characteristics of study participants measured at baseline by recovery status at 12 months and unadjusted odds ratios (row percentage)

Characteristics	Not fully recovered *N* = 264 (68.9%)	Fully recovered *N* = 119 (31.1%)	Unadjusted OR (95% CI)
Centre			
Nottingham	93 (69.9)	40 (30.1)	1.00
Loughborough	67 (67.0)	33 (33.0)	1.15 (0.66, 2.00)
Bristol	87 (73.1)	12 (26.9)	0.86 (0.49, 1.48)
Surrey	17 (54.8)	14 (45.2)	1.91 (0.86, 4.26)
Age			
16–24	24 (63.2)	14 (36.8)	1.00
25–44	48 (67.6)	23 (33.4)	0.82 (0.36, 1.88)
45–64	156 (73.9)	55 (26.1)	0.60 (0.29, 1.25)
65+	36 (57.1)	27 (42.9)	1.29 (0.56, 2.94)
Sex			
Female	140 (68.0)	55 (32.0)	1.00
Male	124 (70.1)	53 (29.9)	0.91 (0.59, 1.40)
Number of psychiatric diagnoses in the past			
0	221 (67.4)	107 (32.6)	1.00
1	27 (75.0)	9 (25.0)	0.69 (0.31, 1.52)
2+	16 (84.2)	3 (15.8)	0.39 (0.11, 1.36)
Depression score	[2]		
Mean (SD)	1.60 (2.47)	0.98 (1.66)	*0.87* ^*a*^ *(0.77, 0.97)*
Median (IQR)	1 (0,2)	0 (0,1)	
Anxiety score	[2]		
Mean (SD)	2.97 (3.35)	2.59 (3.06)	0.96^a^ (0.90, 1.03)
Median (IQR)	2 (0.5)	1 (0.4)	
AUDIT	[10]	[2]	
Mean (SD)	4.31 (4.06)	4.62 (4.10)	1.02^a^ (0.97, 1.07)
Median (IQR)	3 (1.6)	4 (2.6)	
DAST	[3]		
Mean (SD)	0.08 (0.43)	0.04 (0.24)	0.72^a^ (0.35, 1.48)
Median (IQR)	0 (0.0)	0 (0.0)	
Long-standing illness		[2]	
No	197 (68.2)	92 (31.8)	1.00
Yes	67 (72.8)	25 (27.2)	0.80 (0.47, 1.35)
Employment	[2]	[2]	
Paid employment	153 (69.2)	68 (30.8)	1.00
Not in paid employment	29 (87.9)	4 (12.1)	*0.31 (0.11, 0.92)*
Retired	55 (58.5)	39 (41.5)	1.60 (0.97, 2.63)
Other	25 (80.7)	6 (19.4)	0.54 (0.21, 1.38)
Ethnic group	[2]		
White	253 (63.4)	117 (31.6)	1.00
BME	9 (81.8)	2 (18.2)	0.48 (0.10, 2.26)
Deprivation score (IMD)	[3]	[6]	
Mean (SD)	16.2 (13.1)	13.9 (10.7)	0.98^a^ (0.96, 1.00)
Median (IQR)	12.3 (7.0, 21.2)	10.3 (6.5, 18.5)	
Marital status	[2]		
Single	54 (68.4)	25 (31.7)	1.00
Married/partnership	163 (67.6)	78 (32.4)	1.03 (0.60, 1.78)
Divorced/widowed	45 (73.8)	16 (26.2)	0.77 (0.37, 1.61)
Nights in hospital	[9]	[5]	
Mean (SD)	8.1 (6.7)	5.7 (4.1)	*0.91 (0.87, 0.96)*
Median (IQR)	6 (3.10)	5 (3.8)	
Injury severity	[1]		
Minor	6 (37.5)	10 (62.5)	1.00
Moderate	189 (69.2)	84 (30.8)	*0.27 (0.09, 0.76)*
Serious or worse	68 (73.1)	25 (26.9)	*0.22 (0.07, 0.67)*
Number of injuries			
1	125 (67.6)	60 (32.4)	1.00
2	81 (71.7)	32 (38.3)	0.82 (0.49, 1.37)
3 or more	58 (68.2)	27 (31.8)	0.97 (0.56, 1.68)
Body part injured			
Other	20 (64.5)	11 (35.5)	1.00
Upper limb	32 (48.5)	34 (51.5)	1.93 (0.80, 4.66)
Lower limb	187 (76.0)	59 (24.0)	0.57 (0.26, 1.27)
Upper and lower limbs	25 (62.5)	15 (37.5)	1.09 (0.41, 2.89)
Injury mechanism			
Other	15 (57.7)	11 (42.3)	1.00
Falls	177 (68.3)	82 (31.7)	0.63 (0.28, 1.44)
Traffic	54 (73.0)	20 (27.0)	0.51 (0.20, 1.28)
Struck	18 (75.0)	6 (25.0)	0.45 (0.14, 1.52)
Place of injury		[1]	
Other	41 (67.7)	20 (32.3)	1.00
Home	59 (72.0)	23 (28.0)	0.82 (0.40, 1.68)
Work	22 (75.8)	8 (24.2)	0.67 (0.26, 1.75)
Road	76 (67.9)	36 (32.1)	0.99 (0.51, 1.93)
Countryside	37 (72.6)	14 (27.5)	0.79 (0.35, 1.79)
Sports facilities	25 (59.5)	17 (40.5)	1.43 (0.63, 3.22)

^a^Odds ratio per unit increase in score. Percentages may not add up to 100 due to rounding. Statistically significant odds ratios are italicised

Given the loss at follow-up as a sensitivity analysis, we used multiple imputations with chained equations to impute missing values for all 668 participants included at baseline. The imputation model included study centre, age, sex, recovery status and all variables considered in blocks B, C and D above, including those reported at baseline and at 1, 2, 4 and 12 months post-injury. Fifty imputed datasets were generated. Results were combined across the imputed datasets using Rubin’s rules [42]. We also undertook a sensitivity analysis restricting analyses to those with HADS depression subscale scores in the normal range (below 8) at 12 months post-injury to explore whether depression at that time point influenced reporting of recovery.

## Results

### Recruitment, follow-up and recovery

**Fig. 1:**
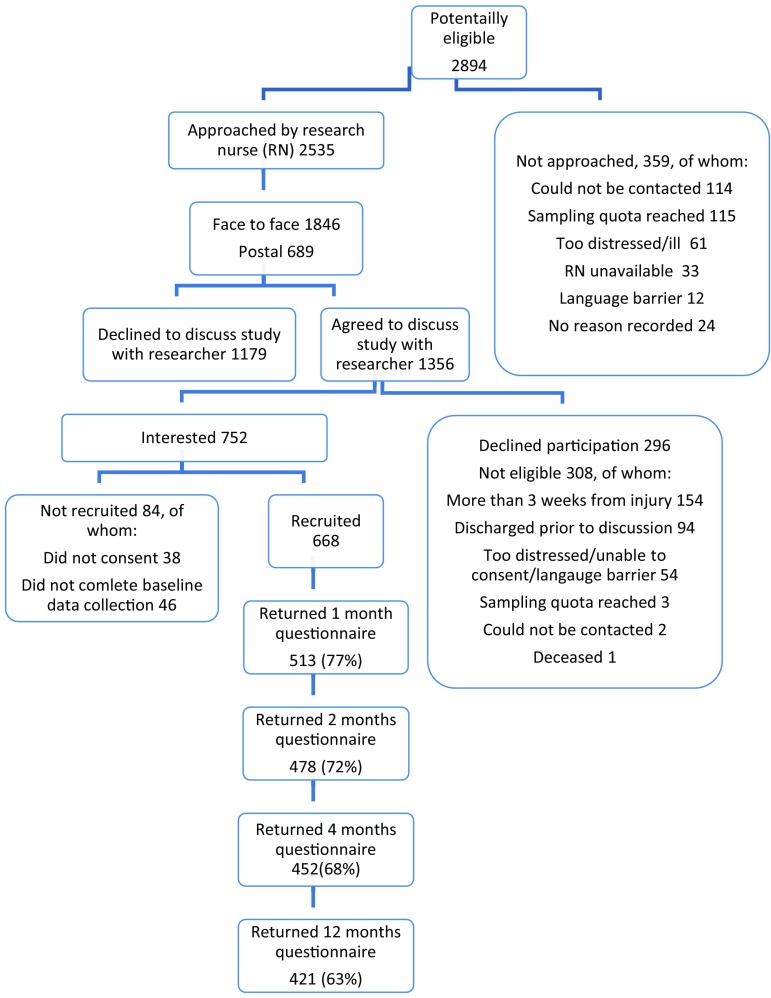
The process of study recruitment and follow-up

Figure 1 shows that 2894 patients were identified as potentially eligible for the study; 2535 were approached to take part in the study, of whom 308 were found to be ineligible. Thirty percent (668/2227) of those approached participated in the study. Forty-seven percent of those approached by the research nurse did not wish to discuss the study with a researcher, and 22% of those that did discuss the study with a researcher did not wish to participate. The most common reasons for ineligibility were length of time since injury and discharge from hospital prior to discussing the study with the researcher.

Of those recruited, 77% were followed up at 1 month and 63% at 12 months. Full recovery was rarely reported before 12 months [1 month: 1% (4/512), 2 months: 1% (7/478), 4 months: 7% (30/451)]. Thirty-one percent (119/383) returning both 1 and 12 month questionnaires reported full recovery at 12 months. Only participants returning both 1 and 12 months questionnaires were included in the main analysis and their characteristics are as follows: 55% were aged 45–64 years, 19% aged 25–44 years and the remaining were under 24 (10%) and over 65 years (16%); 51% were female; 24% had a long-standing illness; 58% were in paid employment, 25% were retired, 7% were not in paid employment and 8% had other employment status (e.g. student); 97% were white; 63% were married, 21% were single and 16% were divorced or widowed; 4% suffered a minor injury, 71% a moderately severe injury and 24% at least a serious injury; 48% had a single injury; 64% injured lower limbs, 17% upper limbs, 11% both upper and lower limbs and 8% injured other body regions; falls caused 68% of injuries, traffic injuries 19%, being struck 6% and other mechanism 7%; the most common locations of injures were on the road (29%), at home (24%), in the countryside (13%) and at sports facilities (11%). Those reporting full recovery at 12 months had significantly higher EQ5D and Health Utilities Index (HUI) scores than those not fully recovered [median (IQR) EQ5D: recovered = 1(0.80, 1), not recovered = 0.73 (0.66, 0.80), *p* < 0.001; median (IQR) HUI: recovered = 0.94 (0.85, 0.97), not recovered = 0.78 (0.57, 0.92), *p* < 0.001]. However, self-reported recovery was not always consistent with functional recovery. One-third (32%; 38/119) of those reporting full recovery had EQ5D scores which were less than 90% of their baseline scores, as did 3% (4/119) for HUI scores. Five percent (13/264) of those not fully recovered had 12 month EQ5D scores more than 10% higher than baseline scores, as did 51% (135/264) for HUI scores.

### Caseness and SCID-DSM-IV criteria over time

Online Table 1 shows the proportions of patients meeting criteria for caseness as defined by cutoffs on the HADS, IES, AUDIT and DAST scales at baseline, 1 and 12 months. There were significant increases 1 month post-injury compared to baseline in the prevalence of depression (15.2 vs 1.4%, *p* < 0.001), anxiety (16.0 vs 4.1%, *p* *<* 0.001) and significant decrease in alcohol problems (12.2 vs 19.6%, *p* < 0.001). Significant increases 12 months post-injury compared to baseline remained for depression (5.7 vs 1.4%, *p* < 0.001) and anxiety (9.7 vs 4.1%, *p* < 0.001) and significant decrease in alcohol problems (19.6 vs 13.3%, *p* < 0.001). Online Table 2 shows the proportions of participants meeting SCID-DSM-IV criteria for mental disorder at baseline amongst those who scored above case level on the HADS, IES, AUDIT and DAST at 1 and 12 months. At both 1 and 12 months post-injury compared to baseline, a significantly higher proportion met the criteria for current major depression (baseline: 1.6%; 1 month: 18.1%; 12 months 17.7% with both *p* < 0.001) and PTSD (baseline 1.6%; 1 month 15.0%; 12 months 11.9%, with *p* values, respectively, *p* < 0.001 and *p* = 0.012). There were also non-significant increases in panic disorder, agoraphobia, specific phobia (usually travel phobia), generalised anxiety disorder, and substance abuse and reduction in alcohol abuse and alcohol dependence compared to baseline.

### Univariate analyses

Table 1 shows the baseline participant characteristics by recovery status at 12 months and results of univariate analyses. A higher depression score at baseline and spending more nights in hospital were associated with significantly reduced odds of recovery. In addition, moderate or serious (or worse) injury compared to minor injury and being unemployed compared to being employed were associated with significantly reduced odds of recovery.

Table 2 shows the participant characteristics measured 1 month post-injury by recovery status at 12 months and results of univariate analyses. A higher depression score, a higher anxiety score, a higher IES score (avoidance subscales), a higher social functioning scale score (indicating poorer social functioning), a higher negative changes in outlook score and a higher pain score were significantly associated with reduced odds of recovery at 12 months. Seeking compensation and involvement in litigation were both significantly associated with reduced odds of recovery.

**Table 2:** Characteristics of study participants measured at 1 month post-injury by recovery status at 12 months and unadjusted odds ratios

Characteristics	Not fully recovered *n* = 264	Fully recovered *n* = 119	Complete case: unadjusted OR (95% CI)	Multiply imputed: unadjusted OR (95% CI)
Depression (score range)^a^				
Quartile 1 (0–3)	69 (53.9)	59 (46.1)	1.00	1.00
Quartile 2 (4–5)	60 (74.1)	21 (25.9)	*0.41 (0.22, 0.75)*	0.58 (0.32, 1.03)
Quartile 3 (6–9)	68 (73.1)	25 (26.9)	*0.43 (0.24, 0.76)*	0.61 (0.36, 1.02)
Quartile 4 (9.3–21)	67 (82.7)	14 (17.3)	*0.24 (0.12, 0.48)*	*0.38 (0.21, 0.67)*
Anxiety score				
Mean (SD)	6.13 (4.41)	4.41 (4.04)	*0.90* ^*b*^ *(0.86, 0.96)*	*0.94 (0.90, 0.99)*
Median (IQR)	5 (3.9)	3 (1.6)		
AUDIT	[4]	[5]		
Mean (SD)	2.89 (3.85)	3.73 (4.12)	1.05^b^ (1.00, 1.11)	1.02 (0.97, 1.07)
Median (IQR)	2 (0.4)	3 (1.4)		
DAST	[4]	[1]		
Mean (SD)	0.02 (0.17)	0.08 (0.48)	1.88^b^ (0.86, 4.08)	1.14 (0.75, 1.73)
Median (IQR)	0 (0.0)	0 (0.0)		
IES avoidance	[1]			
Mean (SD)	8.24 (9.35)	5.39 (6.92)	*0.96* ^*b*^ *(0.93, 0.99)*	0.97 (0.95, 1.00)
Median (IQR)	5 (0.14)	3 (0.10)		
IES intrusion	[1]			
Mean (SD)	8.45 (8.80)	6.43 (7.69)	0.97^b^ (0.94, 1.00)	0.99 (0.96, 1.01)
Median (IQR)	6 (1.4)	3 (0.10)		
SFQ	[1]	[1]		
Mean (SD)	7.94 (3.61)	6.12 (3.26)	*0.85* ^*b*^ *(0.79, 0.91)*	*0.91 (0.85, 0.97)*
Median (IQR)	7 (5.10)	6 (4.8)		
CSS	[1]	[1]		
Mean (SD)	31.90 (6.49)	32.88 (5.69)	1.03^b^ (0.99, 1.06)	1.02 (0.98, 1.05)
Median (IQR)	33 (28.36)	34 (30.37)		
Changes in outlook (+ve)	[1]			
Mean (SD)	19.92 (6.58)	18.94 (6.52)	0.98^b^ (0.95, 1.01)	0.99 (0.96, 1.02)
Median (IQR)	22 (17.25)	20 (14.24)		
Changes in outlook (−ve)	[1]			
Mean (SD),	10.59 (5.42)	8.53 (4.18)	*0.91* ^*b*^ *(0.87, 0.96)*	*0.95 (0.90, 0.99)*
Median (IQR)	9 (6.14)	7 (5.11)		
Life events since injury	[5]	[6]		
No	221 (68.6)	101 (31.4)	1.00	1.00
Yes	38 (76.0)	12 (24.0)	0.69 (0.35, 1.38)	0.90 (0.48, 1.66)
Pain VAS	[3]	[1]		
Mean (SD),	32.41 (21.73)	20.63 (18.77)	*0.97* ^*b*^ *(0.96, 0.98)*	*0.98 (0.97, 0.99)*
Median (IQR)	29 (15.50)	15 (5.31)		
Seeking compensation	[18]	[8]		
No	189 (66.1)	97 (33.9)	1.00	1.00
Yes	57 (80.3)	14 (19.7)	*0.48 (0.25, 0.90)*	0.65 (0.38, 1.13)
Involved in litigation	[3]	[1]		
No	18 (66.5)	110 (33.5)	1.00	1.00
Yes	43 (84.3)	8 (15.7)	0*.37 (0.17, 0.81)*	0.62 (0.32, 1.19)

^a^Depression scores were categorised into quartiles because the relationship with recovery was non-linear

^b^Odds ratio per unit increase in score. Percentages may not add up to 100 due to rounding. Statistically significant odds ratios are italicised

### Multivariable analyses

Table 3 shows the relationships between psychological morbidity at 1 month and recovery at 12 months, adjusted for a priori defined confounders (study centre, age and sex), socio-demographic, psychological and injury characteristics measured at baseline and potential predictors of recovery measured at 1 month. The final model (model D) shows that higher depression scores at 1 month were associated with a lower odds of recovery, as were spending more nights in hospital and greater levels of pain. Deprivation and employment status were not significantly associated with recovery, but are likely to confound the relationship between depression and recovery, as removing them from the model resulted in the odds ratios for depression scores changing by at least 10%. There were no significant interactions between depression score and other predictors in the model.

**Table 3:** Psychological predictors (at 1 month post-injury) of recovery at 12 months, adjusted for confounders, socio-demographic and injury characteristics and other significant predictors (complete case analysis)

Characteristics	Model A: a priori confounders (*n* = 383)	Model B: model A + psychological predictors at 1 month (*n* = 383)	Model C: model B + psychological predictors at 1 month + socio-demographic, psychological and injury characteristics at baseline (*n* = 356)	Model D: model C + other predictors at 1 month (*n* = 353)
	Odds ratio (95% CI)	Odds ratio (95% CI)	Odds ratio (95% CI)	Odds ratio (95% CI)
A priori confounders				
Centre				
Nottingham	1.00	1.00	1.00	1.00
Loughborough	1.14 (0.64, 2.02)	1.16 (0.64, 2.08)	1.22 (0.64, 2.33)	1.22 (0.63, 2.33)
Bristol	0.84 (0.48, 1.47)	0.73 (0.41, 1.31)	0.77 (0.41, 1.47)	0.78 (0.41, 1.48)
Surrey	2.07 (0.92, 4.65)	1.72 (0.74, 4.00)	1.35 (0.53, 3.46)	1.44 (0.56, 3.70)
Age				
16–24	1.00	1.00	1.00	1.00
25–44	0.81 (0.35, 1.87)	0.93 (0.39, 2.18)	0.60 (0.22, 1.66)	0.75 (0.27, 2.12)
45–64	0.58 (0.28, 1.23)	0.61 (0.28, 1.31)	0.35 (0.13, 0.90)	0.43 (0.16, 1.15)
65+	1.30 (0.56, 3.04)	1.30 (0.54, 3.10)	0.36 (0.10, 1.29)	0.45 (0.13, 1.62)
Sex				
Female	1.00	1.00	1.00	1.00
Male	0.88 (0.56, 1.38)	0.82 (0.51, 1.31)	0.88 (0.52, 1.47)	0.82 (0.49, 1.39)
Psychological predictors measured at 1 month post-injury				
Depression score				
Quartile 1 (0–3)		1.00	1.00	1.00
Quartile 2 (4–5)		*0.37 (0.20, 0.69)*	*0.41 (0.20, 0.81)*	*0.46 (0.23, 0.92)*
Quartile 3 (6–9)		*0.42 (0.23, 0.77)*	*0.44 (0.23, 0.87)*	0.57 (0.29, 1.11)
Quartile 4 (9.3–21)		*0.25 (0.13, 0.50)*	*0.24 (0.11, 0.52)*	*0.33 (0.15, 0.73)*
Socio-demographic, psychological and injury characteristics at baseline				
Employment				
In paid employment			1.00	1.00
Not in paid employment			0.34 (0.07, 1.59)	0.35 (0.08, 1.66)
Retired			*2.41 (1.09, 5.35)*	2.02 (0.91, 4.47)
Other			0.35 (0.11, 1.11)	0.38 (0.12, 1.23)
Deprivation (IMD)			1.00 (0.98, 1.02)	1.00 (0.98, 1.03)
Nights in hospital			*0.91 (0.86, 0.97)*	*0.91 (0.86, 0.97)*
Severity				
Minor			1.00	
Moderate			*0.24 (0.06, 0.93)*	
Serious or worse			*0.16 (0.04, 0.69)*	
Other predictors measured at 1 month post-injury				
Pain visual analogue scale				*0.98 (0.97, 0.99)*

Only predictors significant in models or which changed the odds ratios for at least one depression score quartile at 1 month by >10% are shown. Statistically significant odds ratios are italicised

Online Table 3 shows participant characteristics comparing those who did and did not return both the 1 and 12 month questionnaires. Those returning both questionnaires were more likely to come from study centres other than Nottingham, be older, female, retired, married/in a civil partnership, of a white ethnic group, live in a less deprived area and have at least a moderately severe injury. They reported fewer alcohol or drug problems at baseline, fewer drug-related problems and lower pain scores at 1 month.

Online Table 4 shows the results of multivariable analysis using multiply imputed data. The findings are similar to those from the complete case analysis. The reduction in the odds of recovery associated with depression was less marked than in the complete case analysis, but remained significant for those with the highest quartile of scores compared to those with the lowest quartile of scores. An increasing number of nights in hospital remained significantly associated with a reduced odds of recovery, with associations being slightly less marked than in the complete case analysis. The relationship between pain and self-reported recovery was smaller in the multiple imputation analysis and of borderline statistical significance.

Online Table 5 shows the results of the sensitivity analysis restricting the multivariable analysis to those with HADS depression subscale scores in the “normal” range at 12 months. Findings were very similar to the complete case analysis.

## Discussion

### Main findings

The outcome for most study participants was poor, with only one-third reporting a full recovery 12 months after the injury. Depression (15%) and anxiety (16%) (as assessed by the HADS) were common 1 month post-injury and although less prevalent at 12 months post-injury, 6% still reported depression and 10% reported anxiety. The number of participants meeting the case definition for psychiatric disorders increased following the injury at 1 month and remained higher than pre-injury at 12 months. Those fully recovered had significantly higher health status scores than those not fully recovered, but health status measures were not always consistent with self-reported recovery, highlighting the importance of using self-reported recovery as an outcome measure. Higher depression scores at 1 month were associated with a lower odds of self-reported recovery at 12 months, as were spending more nights in hospital and greater levels of pain

### Strength and limitations

Unlike many other studies, we used subjectively defined self-reported recovery as the outcome of interest, so adding to the body of knowledge about psychological morbidity and functional or health status measures of recovery. Our study also addressed some of the limitations of previous studies by investigating a general injury population with different types of injuries of varying severity, using a range of psychological predictors of recovery, adjusting for a wide range of potential confounders (injury characteristics, socio-demographic, physical, occupational and socio-legal factors), having a larger sample size than some studies, achieving an acceptable follow-up rate and taking account of losses to follow-up and missing data using multiple imputations.

Of the eligible patients (2227) who approached to take part in the study 30% (668) participated. It is possible that selection bias occurred if participation was related to recovery. During recruitment and follow-up data collection, the study aims were described as identifying the impact of injury in general, without emphasis on psychological factors or pain, to try and minimise overreporting of those variables and overestimation of their effect on recovery. Our follow-up rate of 63% at 12 months was lower than that in some studies [21] and higher than in others [43] and may be related to the number of follow-up questionnaires used. There were significant differences in characteristics between those returning both the 1 and 12 month questionnaires and those who did not. Our multiple imputation analysis supports our findings from the complete case analysis on the role of depression and nights in hospital in predicting recovery. This suggests our analysis is robust to missing data, although the associations were less marked than in the complete case analysis.

Although we recruited participants with a wide range of injuries, the numbers with some types of injuries were small and analysis was restricted to broad injury groupings. While we measured a wide range of confounding factors, some residual confounding may still be present. Black and ethnic minority groups were underrepresented which may limit generalisability of our findings for these groups. In addition, younger adults, particularly males, were underrepresented in our study at follow-up. Since alcohol and non-alcohol substance use disorders are more common in young men, the influence of these problems on recovery may be underestimated. As some mental disorder is present in people scoring below cutoffs for caseness and SCIDs are only undertaken in those reaching case level, SCID mental disorders at follow-up are likely to be underestimated. New mental disorder requiring a duration of greater than 1 month for diagnosis, e.g. substance abuse and dependence or generalised anxiety disorder would not have been captured by SCIDs completed 1 month post-injury. However, none of these issues with the measurement of mental disorder using psychiatric interview detract from the results of our analysis exploring the effects of self-reported symptoms of depression, anxiety, PTSD or substance use on self-reported recovery. The SCID interview data confirm that clinically important depression, anxiety and PTSD prior to injury were as common in study participants as in the general population, alcohol use disorders were somewhat higher and all were more prevalent 12 months post-injury. Despite the issues outlined, our study was able to account for a number of important factors that previous literature has shown to be important in predicting recovery, and identify psychological factors that remain important after other factors have been accounted for.

### Comparisons with previous research

To our knowledge, there are no published studies exploring the relationship between psychological morbidity and self-reported recovery in a general injury population with which to compare our findings. Two previous studies used self-reported recovery measures, but neither explored psychological factors associated with recovery and both found higher recovery rates than in our study, probably due to inclusion of more minor injuries than in our study [3, 44]. Our study highlights the importance of depression and pain, two modifiable factors, in predicting self-reported recovery, adding to our knowledge that these factors are important in predicting functional recovery. The relationship between pain and depression is complex with both being shown to have a strong effect on each other over time [45]. Previous studies show depression or a combination of PTSD and depression predicted poorer quality of well-being [16] and depression predicted poorer functional outcomes [8, 43, 46]; depression, anxiety or travel anxiety predicted physical recovery [4], and psychological distress and PTSD predicted disability [17, 47]. Consistent with our findings, previous studies also show pain [8, 9, 17, 48] and length of stay in the hospital [9] to be associated with functional recovery.

The rates of psychiatric disorder in the 2 years prior to study participation are comparable to the population in the catchment areas of our study sites [49]. Therefore, the effect of depression on recovery is largely unrelated to pre-injury mental health problems. Unlike previous research, our study did not show PTSD to predict recovery. Given the high rates of PTSD symptoms (measured using the IES) at 1 and 12 months and PTSD (measured using the SCID) by 12 months, it is likely that PTSD psychopathology contributed to the effect of depression on recovery since these conditions commonly co-exist and depression symptoms are a common feature of PTSD.

### Implications for practice

Depression and pain at 1 month post-injury are both common and important modifiable predictors of recovery at 12 months post-injury amongst a general injury population. It is important for injured patients to understand the relationship between depression, pain and recovery and to seek advice and support for these problems. Primary and secondary health-care services need to identify, clinically assess and manage persisting depression at 1 month, and measure and adequately control persisting pain, as part of post-injury care and rehabilitation. The relationship between pain and depression is complex, and each may have multiple contributory factors, but both need addressing in post-injury care. Health professionals routinely treating people with unintentional injuries are not mental health experts. It would be useful if they can identify patients at risk of poor recovery using standard self-report measures of psychological health and pain, help patients manage these conditions and refer to appropriate services as necessary [50, 51]. In addition, our study shows a simple and routinely available measure, such as the number of nights in hospital, can highlight those at risk of poor recovery.

### Implications for research

Our study focussed on the impact of early psychological morbidity on recovery from injury, but given the prevalence of depression, anxiety and symptoms of post-traumatic distress 12 months post-injury, future studies should explore the impact of persistent psychological morbidity on recovery. Future studies exploring the short- and longer-term impact of injuries should include measures of psychological morbidity and pain. Studies exploring psychological morbidity and outcomes (such as self-reported recovery, return to work and quality of life) need to consider adjustment for pain and psychological factors. Future recruitment strategies should focus on increasing participation of 16- to 24-year-olds and ethnic minorities.

## Electronic supplementary material

Below is the link to the electronic supplementary material.

Supplementary material 1 (DOCX 47 kb)
