# Supplementary material for: The impact of psychological factors on recovery from injury: a multicentre cohort study
Source: Soc Psychiatry Psychiatr Epidemiol. 2016 Nov 1;52(7):855–66. doi: 10.1007/s00127-016-1299-z (PMC5504249; doi:10.1007/s00127-016-1299-z)
Supplement: Supplementary file 1 — Supplementary material 1 (DOCX 47 kb) [file 127_2016_1299_MOESM1_ESM.docx]

**Supplementary materials**

Online Table 1: Proportion of participants meeting definitions of caseness for psychological measures at baseline, 1 month and 12 months follow-up.

| Psychological measure | Meets caseness definition (%) | | |
| --- | --- | --- | --- |
|  | Baseline (N=513)* | 1 month (N=513) | 12 months (N=383) |
| Depression (HADS depression score ≥11) | 7(1.4)[2] | 78(15.2)[1]** | 22(5.7)** |
| Anxiety (HADS anxiety score ≥ 11) | 21(4.1) [2] | 82(16.0) [1]** | 37(9.7) ** |
| PTSD (IES score ≥26; moderate or severe) | N/A | 126(24.7)[3] | 68(17.8)[2] |
| Alcohol (score ≥8; medium or high) | 98(19.6)[14]** | 61(12.2)[13]** | 50(13.3)[9] ** |
| Drugs (DAST score ≥3; moderate or severe). | 7(1.4%)[4] | 4(0.8)[7] | 2(0.5)[8] |
| Meets at least one of the above 5 psychological measures case definitions. | 116(23.3)[[16]] | 194(38.3)[[6]] | 120(31.9)[[6]] |

*Analysis restricted to those participants returning 1 month follow-up ( n=513) as these are the sample used for analyses presented in this paper. []missing values . [[]]data were missing on one or more of the 5 variables and case definitions were not met for the other measures. **Significant change from baseline at p<0.001

Online Table 2: Proportion of participants meeting criteria for psychiatric disorders from SCID interview at baseline, 1 month and 12 months follow-up.

| Psychological measure | Meets caseness definition (%) | | |
| --- | --- | --- | --- |
|  | Baseline *  Completed SCIDs N=513 | 1 Month  SCIDs required based on screening questionnaire N=264**  Completed SCIDs N=193 (73%)*** | 12 months  SCIDs required based on screening questionnaire N=147  Completed SCIDs N=84 (57%) |
| Current Major Depression Episode | 8(1.6) | 35(18.1)**** | 14(17.7)**** |
| Past major depressive episode | 27(5.3) | N/A | N/A |
| Dysthymic disorder | 9(1.8) | N/A | N/A |
| Panic Disorder | 9(1.8) | 6(3.1) | 9(10.7) |
| Panic Disorder with agoraphobia | 4(0.8) | 1(0.5) | 2(2.4) |
| Agoraphobia without history of panic disorder | 2(0.4) | 3(1.6) | 3(3.6) |
| Social phobia | 10(1.9) | 0(0.0) | 1(1.2) |
| Specific phobia | 10(1.9) | 5(2.6) | 3(3.6) |
| Obsessions and Compulsion (OCD) | 5(1.0) | 1(0.5) | 0(0.0) |
| Generalised anxiety disorder | 7(1.4) | 4(2.0) | 4(4.8) |
| PTSD | 8(1.6) | 29(15.0)**** | 10(11.9)***** |
| Alcohol abuse | 15(2.9) | 4(2.0) | 4(4.8) |
| Alcohol dependence | 11(2.1) | 2(1.0) | 3(3.6) |
| Substance abuse | 2(0.4) | 3(1.6) | 1(1.2) |
| Substance dependence | 2(0.4) | 0(0.0) | 0(0.0) |

* Analysis restricted to those participants returning 1 month follow-up ( n=513) as these are the sample used for analyses presented in this paper. ****** Only those participants scoring one or more of the following cut-offs required SCID interviews: scores of borderline or caseness in HADS depression and HADS anxiety, moderate or severe in IES and DAST, medium or high in AUDIT. ***People who did not have their SCID interview done were dropped from analysis. ****Significant change from baseline at p<0.001. ****Significant change from baseline at p<0.05

**Online table 3. Characteristics measured at baseline and one month in those who returned 1 and 12 month questionnaires compared with those who did not (row percentages)**

| ***Characteristics measured at baseline*** | | | | |
| --- | --- | --- | --- | --- |
|  | **Did not return both the 1 and 12 month questionnaires (n=284)** | **Returned 1 and 12 month questionnaires (n=384)** | **P value** | |
| Centre  Nottingham  Loughborough  Bristol  Surrey | 145 (52.2)  66 (39.5)  55 (31.6)  18 (36.7) | 133 (47.8)  101 (60.5)  119 (68.4)  31 (63.3) | P<0.01 | |
| Age  16-24  25-44  45-64  65+ | 58 (60.4)  106 (59.6)  99 (31.9)  21 (25.0) | 38 (39.6)  72 (40.5)  211 (68.1)  63 (75.0) | P<0.01 | |
| Sex  Female  Male | 110 (34.8)  174 (49.4) | 206 (65.2)  178 (50.6) | P<0.01 | |
| Number of psychiatric diagnoses in past  0  1  2+ | 227 (40.8)  30 (45.5)  27 (58.7) | 329 (59.2)  36 (55.6)  19 (41.3) | P=0.06 | |
| Depression score  mean (SD)  median (IQR) | 1.9 (3.1)  0 (0,3) | [2]  1.4 (2.3)  0 (0,2) | P=0.32 | |
| Anxiety score  mean (SD)  median (IQR) | 3.3 (4.0)  2 (0,5) | [2]  2.9 (3.3)  2 (0,5) | P=0.62 | |
| AUDIT  mean (SD)  median (IQR) | [8]  5.9 (5.8)  4 (2,8) | [3]  4.4 (4.1)  4 (1,6) | P<0.01 | |
| DAST  mean (SD)  median (IQR) | [4]  0.4 (1.3)  0 (0,0) | [3]  0.1 (0.4)  0 (0,0) | P<0.01 | |
| Long standing illness  No  Yes | [4]  208 (41.8)  72 (43.9) | [2]  290 (58.2)  92 (56.1) | P=0.63 | |
| Employment  Paid employment  Not in paid employment  Retired  Other | [4]  171 (43.5)  43 (56.6)  36 (27.7)  30 (49.2) | [4]  222 (56.5)  33 (43.4)  94 (72.3)  31 (50.8) | P<0.01 | |
| Ethnic group  White  BME | 263 (41.5)  21 (65.6) | [2]  371 (58.5)  11 (34.4) | P<0.01 | |
| Deprivation (IMD)  mean (SD)  median (IQR) | [8]  20.7 (15.1)  15.5 (9.1, 29.0) | [9]  15.5 (12.4)  11.4 (6.9, 20.0) | P<0.01 | |
| Marital status  Single  Married/partnership  Divorced/widowed | [3]  110 (58.2)  118 (32.8)  53 (46.5) | [2]  79 (41.8)  242 (67.2)  61 (53.5) | P<0.01 | |
| Nights in hospital  Mean (SD)  Median (IQR) | 7.3 (5.7)  6 (3,10) | 7.3 (6.1)  6 (3,9) | P=0.81 | |
| Injury severity  Minor  Moderate  Serious or worse | [1]  28 (63.6)  197 (41.8)  58 (38.6) | [1]  16 (36.4)  274 (58.2)  93 (61.6) | P=0.01 | |
| Number of injuries  1  2  3 or more | 132 (41.6)  98 (46.5)  54 (38.6) | 185 (58.4)  113 (53.6)  86 (61.4) | P=0.31 | |
| Body part injured  Other  Upper limb  Lower limb  Upper and lower limbs | 29 (48.3)  51 (43.6)  183 (42.7)  21 (33.9) | 31 (51.7)  66 (56.4)  246 (57.3)  41 (66.1) | P=0.43 | |
| Injury mechanism  Other  Falls  Traffic  Struck | 28 (51.9)  166 (39.1)  67 (47.2)  23 (48.9) | 26 (48.2)  259 (60.9)  75 (52.8)  24 (51.1) | P=0.11 | |
| Place of injury  Other  Home  Work  Road  Countryside  Sports facilities | 41 (39.8)  60 (42.3)  29 (46.0)  88 (44.0)  25 (32.9)  41 (49.4) | 62 (60.2)  82 (57.8)  34 (54.0)  112 (56.0)  51 (67.1)  42 (50.6) | P=0.38 | |
| ***Characteristics measured at 1 month*** | | | | |
|  | **Returned 1 month questionnaire but did not return 12 month questionnaire (n=129)** | **Returned 1 and 12 month questionnaires (n=384)** | | **P value** |
| Depression score  Mean (SD)  Median (IQR) | [1]  6.2 (4.4)  5 (2,9) | 6.0 (4.3)  5 (3,9) | | P=0.70 |
| Anxiety score  Mean (SD)  Median (IQR) | [1]  6.4 (4.4)  6 (2.7, 10) | 5.6 (4.4)  5 (2,8) | | P=0.06 |
| AUDIT  Mean (SD)  Median (IQR) | [4]  5.9 (5.8)  4 (2,8) | [9]  3.1 (3.9)  2 (0,4) | | P=0.06 |
| DAST  Mean (SD)  Median (IQR) | [2]  0.1 (0.5)  0 (0,0) | [5]  0.0 (0.3)  0 (0,0) | | P<0.01 |
| IES avoidance  Mean (SD),  Median (IQR) | [2]  9.0 (9.6)  6 (0,16) | [1]  7.3 (8.8)  4 (0,12) | | P=0.08 |
| IES intrusion  Mean (SD),  Median (IQR) | [2]  9.6 (9.8)  6 (1,16) | [1]  7.8 (8.5)  5 (0,12) | | P=0.08 |
| SFQ  Mean (SD)  Median (IQR) | [3]  7.9 (3.7)  8 (5,10) | [2]  7.4 (3.6)  7 (5,9) | | P=0.16 |
| CSS  Mean (SD)  Median (IQR) | [3]  31.5 (5.5)  33 (28,36) | [2]  32.2 (6.3)  34 (28, 36) | | P=0.09 |
| Changes in outlook (+)  Mean (SD)  Median (IQR) | [3]  19.0 (6.3)  21 (14, 24) | [1]  19.6 (6.6)  21 (16, 24) | | P=0.27 |
| Changes in outlook  (-)  Mean (SD),  Median (IQR) | [2]  10.0 (5.0)  9 (5,13) | [2]  9.9 (5.1)  9 (5,12) | | P=0.63 |
| Life events since injury  No  Yes | [3]  104 (24.4)  22 (30.1) | [11]  322 (75.6)  51 (69.9) | | P=0.30 |
| Pain VAS  Mean (SD),  Median (IQR) | 34.8 (25.0)  28 (15, 52) | [4]  28.7 (21.5)  24 (11,47) | | P=0.02 |
| Seeking compensation  No  Yes | [5]  98 (25.5)  26 (26.8) | [26]  287 (74.5)  71 (73.2) | | P=0.79 |
| Involved in litigation  No  Yes | [3]  106 (24.4)  20 (28.2) | [4]  329 (75.6)  51 (71.8) | | P=0.49 |

Percentages may not add up to 100 due to rounding

**Online Table 4. Psychological predictors (at 1 month post-injury) of recovery at 12 months, adjusted for confounders, socio-demographic and injury characteristics and other significant predictors (analysis of multiply imputed data for all 668 participants at baseline).**

| **Characteristics** | **Model A**  **(A priori confounders)** | **Model B**  **(Model A + psychological predictors at 1 month)** | **Model C**  **(Model B + psychological predictors at 1 month + socio-demographic, psychological and injury characteristics at baseline)** | **Model D**  **(Model C + other predictors at 1 month)** |
| --- | --- | --- | --- | --- |
|  | **Odds ratio (95% CI)** | **Odds ratio (95% CI)** | **Odds ratio (95% CI)** | **Odds ratio (95% CI)** |
| ***A priori confounders*** | | | | |
| Centre:  Nottingham  Loughborough  Bristol  Surrey | 1.00  1.23 (0.74 , 2.06)  0.80 (0.48 , 1.34)  2.20 (1.04 , 4.68) | 1.00  1.20 (0.71 , 2.03)  0.77 (0.45 , 1.30)  2.03 (0.94 , 4.38) | 1.00  1.21 (0.69 , 2.10)  0.77 (0.45 , 1.33)  1.75 (0.77 , 3.96) | 1.00  1.19 (0.69, 2.06)  0.75 (0.43, 1.31)  1.74 (0.77, 3.92) |
| Age:  16-24  25-44  45-64  65+ | 1.00  0.90 (0.45 , 1.79)  0.67 (0.34 , 1.34)  1.25 (0.54 , 2.87) | 1.00  0.92 (0.45 , 1.88)  0.67 (0.33 , 1.36)  1.19 (0.51 , 2.76) | 1.00  0.79 (0.36 , 1.69)  0.54 (0.25 , 1.19)  0.71 (0.25 , 2.05) | 1.00  0.78 (0.36, 1.72)  0.52 (0.23, 1.17)  0.63 (0.22, 1.82) |
| Sex:  Female  Male | 1.00  0.99 (0.66 , 1.48) | 1.00  0.91 (0.60 , 1.39) | 1.00  0.95 (0.61 , 1.47) | 1.00  0.95 (0.61, 1.47) |
| ***Psychological predictors measured at 1 month post-injury*** | | | | |
| Depression:  Quartile 1 (0-3)  Quartile 2 (4-5)  Quartile 3 (6-9)  Quartile 4 (9.3-21) |  | 1.00  0.55 (0.30 , 0.99)  0.62 (0.36 , 1.05)  0.39 (0.22 , 0.70) | 1.00  0.54 (0.29 , 1.02)  0.63 (0.36 , 1.11)  0.40 (0.22 , 0.75) | 1.00  0.59 (0.32, 1.10)  0.73 (0.41, 1.30)  0.53 (0.28, 1.00) |
| ***Socio-demographic, psychological and injury characteristics at baseline*** | | | | |
| Employment:  In paid employment  Not in paid employment  Retired  Other |  |  | 1.00  0.75 (0.30 , 1.85)  1.73 (0.87 , 3.44)  0.50 (0.19 , 1.29) | 1.00  0.79 (0.32, 1.97)  1.72 (0.86, 3.42)  0.48 (0.18, 1.27) |
| Deprivation (IMD) |  |  | 0.99 (0.98 , 1.01) | 1.00 (0.98, 1.01) |
| Nights in hospital |  |  | 0.95 (0.90 , 0.99) | 0.94 (0.90, 0.99) |
| Injury severity:  Minor  Moderate  Serious or worse |  |  | 1.00  0.70 (0.26 , 1.86)  0.56 (0.19 , 1.64) |  |
| ***Other predictors measured at 1 month post-injury*** | | | | |
| Pain visual analogue scale |  |  |  | 0.99 (0.97, 1.00) |

Statistically significant odds ratios are highlighted.

**Online table 5: Sensitivity analysis of final model for psychological predictors (at 1 month post-injury) of recovery at 12 months, adjusted for confounders, socio-demographic and injury characteristics and other significant predictors restricted to participants with HADS depression subscale scores <8 at 12 months.**

| **Characteristics** | **Final model (Model D) n=315** |
| --- | --- |
|  | **Odds ratio (95% CI)** |
| ***A priori confounders*** | |
| Centre:  Nottingham  Loughborough  Bristol  Surrey | 1.00  1.35 (0.69, 2.66)  0.78 (0.40, 1.52)  1.47 (0.57, 3.81) |
| Age:  16-24  25-44  45-64  65+ | 1.00  0.71 (0.25, 2.05)  0.41 (0.15, 1.13)  0.44 (0.12, 1.66) |
| Sex:  Female  Male | 1.00  0.88 (0.51, 1.52) |
| ***Psychological predictors measured at 1 month post-injury*** | |
| Depression:  Quartile 1 (0-3)  Quartile 2 (4-5)  Quartile 3 (6-9)  Quartile 4 (9.3-21) | 1.00  0.48 (0.24, 0.97)  0.59 (.30, 1.18)  0.37 (0.16, 0.84) |
| ***Socio-demographic, psychological and injury characteristics at baseline*** | |
| Employment:  In paid employment  Not in paid employment  Retired  Other | 1.00  0.44 (0.09, 2.16)  2.19 (0.95, 5.02)  0.27 (0.08, 0.99) |
| Deprivation (IMD) | 1.00 (0.98, 1.03) |
| Nights in hospital | 0.92 (0.86, 0.98) |
| ***Other predictors measured at 1 month post-injury*** | |
| Pain visual analogue scale | 0.98 (0.96, 0.99) |

Statistically significant odds ratios are highlighted.
